# Unemployment and disability pension-an 18-year follow-up study of a 40-year-old population in a Norwegian county

**DOI:** 10.1186/1471-2458-12-148

**Published:** 2012-02-28

**Authors:** Morten Støver, Kristine Pape, Roar Johnsen, Nils Fleten, Erik R Sund, Bjørgulf Claussen, Johan H Bjørngaard

**Affiliations:** 1 Department of Public Health and General Practice, Faculty of Medicine, Norwegian University of Science and Technology, MTFS, 7491 Trondheim, Norway; 2 Department of Community Medicine, Faculty of Health Sciences, University of Tromsø, 9037 Tromsø, Norway; 3 Northern Norway Regional Health Authority, Tromsø 9038 Tromsø, Norway; 4 Department of General Practice and Community Medicine, University of Oslo, 0318 Oslo, Norway; 5 St. Olav's University Hospital Trondheim, Forensic Department and Research Centre Brøset, 7440 Trondheim, Norway; 6 NTNU, Department of Public Health and General Practice, Faculty of Medicine, Postboks 8905, MTFS, 7491 Trondheim, Norway

**Keywords:** Disability benefit, Disability pension, Unemployment, Work disability, Multilevel modelling

## Abstract

**Background:**

This study explored the association of unemployment and an increased risk of receiving disability pension, and the possibility that this risk is attributed to municipality-specific characteristics.

**Methods:**

A cohort of 7,985 40-42 year olds was followed for 18 years in national registers, identifying new episodes of unemployment and cases of disability pension. The association between an unemployment period and disability pension in the subsequent year was estimated using discrete time multilevel logistic regressions and clustering individuals by municipality. The association between unemployment and disability pension was adjusted for age in the follow up-period, sex, baseline health status, health behaviour and education level. A conditional intra-class correlation coefficient (ICC) was estimated as a measure of inter-municipality variance.

**Results:**

In the follow-up period, 2784 (35%) of the participants were granted disability pension. The crude odds ratio for receiving disability pension after unemployment (adjusted for age in follow-up period and sex only) was 1.42 (95% CI 1.1-1.8). Adjusting for baseline health indicators reduced the odds ratio of unemployment to 1.33 (CI 1.1-1.7). A fully adjusted model, including education level, further reduced the odds ratio of unemployment to 1.25 (CI 1.00-1.6). The ICC of the municipality level was approximately 2%.

**Conclusions:**

Becoming unemployed increased the risk of receiving subsequent disability pension. However, adjusting for baseline health status, health behaviour and education attenuated this impact considerably. The multilevel analysis indicated that a minor, yet statistically significant, proportion of the risk of disability pension can be attributed to the municipality of residence.

## Background

When a person's ability to work is hampered by disease, the medically based disability pension is a cornerstone in the economic compensation for lost income. Occupational life is important for self-identity, health and well-being [1,2], and the association between unemployment and poor health is well documented [3,4]. Furthermore, unemployment and organizational downsizing have been associated with subsequent disability pensions [5-8]. Past experience indicates that economic downturns affect disadvantaged people greater than others and increases the number of unemployed disabled workers [9]. The recent economic recession highlights the need for increased attention to prevent further inflows from unemployment into disability pension.

Although unemployment and poor health status are associated, it remains unclear whether unemployment leads to poor health and disability, or if people with poorer health are more vulnerable to labour market fluctuations, and thus more likely to become unemployed. Some studies suggest that job loss, and the subsequent unemployment period, leads to poor health [10-12]. However, the research is not conclusive [13], and other studies suggest that people with poor health have a higher risk of unemployment [14,15]. Regardless of unemployment being a cause or consequence of poor health, both suggest an explanation for the growing number of people receiving disability pensions; work disability does not arise from health impairments alone, but rather it arises from the combination of health impairments and poor employment opportunities [16].

The risk of unemployment is closely connected to local labour market fluctuations. Hence, any study of the association between unemployment and work disability should take into account possible geographical outcome variations. Multilevel analysis with people nested by municipality is a suitable analytical tool to assess this outcome, but the research on geographical differences in disability pensions within a multilevel analytical framework is limited. However, studies on work disability suggest that geographical differences are related to level of urbanization [17,18], municipality and county deprivation [19], as well as variations in praxis of rejecting applicants [20].

By following a cohort of 40- to 42-year-old men and women for a period of 18 years, we have explored the association of unemployment and an increased risk of being granted disability pension and the influence of health, sex, education, age and location of residence on this risk.

## Methods

The data were a part of the National Health Screening Service in Norway and were collected in the Nordland County from August 1988 to March 1989. Individual-level information was obtained from a database of national insurance, created by Statistics Norway and the Norway National Insurance Service. Follow-up time was from January 1, 1992 to December 31, 2007. The study was approved by the Regional Committee for Medical Research Ethics (2009/205-4).

Nordland County is one of 19 counties and is situated in the northern part of Norway. In 1990, Nordland County had 45 municipalities and 239,532 inhabitants. In Statistics Norway's categorization, expressed in terms of the relative distribution of industries in relation to the working population residing in the municipalities in 1990, Nordland County had municipalities where the main industries were fishing, agriculture, manufacturing and services. The diverse types of industries in the municipalities were likely affected differently by business fluctuations during the follow-up period.

### Disability pension

Disability pension was established to ensure sufficient income for people whose earning ability is permanently impaired by at least 50% due to illness or injury. Although each insurance office can exercise some discretion in their decisions, and thus be more lenient to people who have obvious problems finding new jobs, the law requires a medical diagnosis. In this study, the dependent variable was the first day of work disability, defined as the time when a person's earning ability was permanently reduced. In most cases, this date represents the first day of long-term sickness benefits for persons who were later granted a disability pension. Data on new incidents of disability pensions were available from January 1, 1992, and covered all cases of disability pensions in Norway. No cases were missed in this period as firm and private disability insurance is always supplementary to the national pension.

### Unemployment

The impact of unemployment was hypothesized to influence the subsequent risk of disability pension with some induction time. Hence, assessing work disability after unemployment was done as a time-varying covariate with a one-year time lag, meaning the risk of work disability is measured one year after becoming unemployed. Participants were classified as unemployed the year they started an unemployment period. With sensitivity analyses, we also tested models without a time lag of unemployment and with a two-year time lag. Data were obtained from the national insurance register.

### Health measures

Baseline information on different aspects of health was used to adjust for health impairment prior to unemployment. A summated index of the number of chronic illnesses included the following conditions: myocardial infarction, angina pectoris, stroke/cerebral infarction, diabetes, high blood pressure, chronic bronchitis, arthritis, Bechterew's disease, cancer, epilepsy, migraine and gastro-intestinal problems. Self-rated health status was assessed by the question, "what is your health condition like?" The question had four answer categories: "Very good", "Good", "Fair" and "Poor". Depression was assessed by the question, "have you been sad or depressed the last 14 days?" The four answer categories ranged from "almost all the time" to "never or rarely". Headache and pains in the neck and shoulders were measured with a four-point scale, ranging from "never/rarely" to "daily". Alcohol use was assessed with a four-point scale, ranging from "non-drinker" to "daily drinker" Smoking was assessed with a three-point scale with the responses of "non-smoker," "former smoker" and "smoker".

### Socio-demographic characteristics

The age of the participants was between 40-42 years at baseline. Education level was used as a measure of socioeconomic status and included the three categories, "primary school", "high school" and "college/university".

### Statistics

The association between unemployment and disability pension was estimated with discrete time multilevel logistic regressions with individuals nested by municipality of residence. In a discrete time logistic regression analysis, time is treated as intervals, and the risk of disability pension (event) is measured within each interval, given that the event has not occurred before [21]. We used one-year intervals that corresponded with calendar years. The risk of receiving disability pension is closely related to age [22], and therefore, we used age during follow-up period and age-squared to assess the combination effect of age and follow-up period.

In order to explore the impact of individual municipalities, we estimated a *conditional Intra- class correlation coefficient *(ICC) [21]. For the present study, the ICC provides an estimate of the relative importance of the municipality location on an individual's propensity to receive disability pension.

The association between unemployment and subsequent disability was performed in three models. Model 1 was adjusted only for age (i.e., age and period) and sex. In Model 2, we also included baseline health status, health behaviour (as measured by alcohol and smoking behaviour). In Model 3, education was added to Model 2. The precision of the estimates was represented by 95% confidence intervals (CI). The analyses were limited to the participants with complete information in all study variables (5,834). All analyses were conducted using STATA 11 software (StataCorp LP, Texas, USA).

### Effect measure modification analysis

We tested statistical interactions among the variables to investigate the effects of age in follow-up, sex and level of education on the unemployment-disability pension odds ratio.

## Results

### Descriptive statistics

A total of 4,302 men and 4,310 women attended the screening, an attendance rate of 78% and 86% for women, respectively [23]. Of the 10,497 people eligible for the survey, 990 were excluded because they received disability pension before start of follow-up. A total of 1,522 (16%) of the remaining persons did not answer the questionnaires, leaving 7,985 participants for follow-up. Participants were followed from January 1, 1992, until December 31, 2007. Follow-up was censored at death or emigration. Altogether, 480 died or emigrated during follow-up.

Descriptive statistics are provided in Table 1. A total of 2,784 (34.9%) of the participants were granted disability pension in the follow-up period.

**Table 1:** Numbers of persons included in descriptive analysis with and without disability pension during follow-up

	*N (%)*	*Disability p (%)*	*No Disability p (%)*
Total	7,985	2,784 (34.9)	5,201 (65.1)
Men	4,097 (51.3)	1,185 (42.6)	2,912 (56.0)
Women	3,888 (48.7)	1,599 (57.4)	2,289 (44.0)
Been unemployed in follow-up	2,417 (30.3)	935 (33.6)	1,482 (28.5)
Chronic illness			
None	3,833 (48.0)	1,307 (47.0)	2,526 (48.6)
1	1,700 (21.3)	526 (18.9)	1,174 (22.6)
2 or more	2,458 (30.7)	951 (34.1)	1,501 (28.8)
Self rated health			
Fair/poor	781 (11.5)	435 (18.5)	346 (7.8)
Very good/good	6,034 (88.5)	1,921 (81.5)	4.113 (92.2)
Headache			
Never/rarely, once or several times per month	6,129 (91.4)	1,996 (86.8)	4,133 (93.8)
Once or several times per week, daily	577 (8.6)	303 (13.2)	274 (6.2)
Pain in neck or shoulder			
Never/rarely, once or several times per month	5,305 (79.9)	1,616 (70.1)	3,689 (84.5)
Once or several times per week, daily	1,335 (20.1)	663 (29.9)	672 (15.5)
Depression			
Never/rarely	4,149 (61.5)	1,293 (55.3)	2,856 (64.9)
Often/almost all the time	2,593 (38.5)	1,045 (44.7)	1,548 (35.1)
Health behaviour			
Non-smoker	2,264 (28.4)	635 (22.8)	1,629 (31.3)
Former smoker	2,063 (25.8)	660 (23.8)	1,403 (27.0)
Smoker	3,657 (45.8)	1,488 (53.5)	2,169 (41.7)
Non-drinker	2,570 (40.9)	916 (43.0)	1,654 (39.9)
Drinking up to 1-2 times per month	3,439 (54.8)	1109 (52.0)	2,330 (56.2)
Drinking more than once a week/daily	267 (4.3)	106 (5.0)	161 (3.9)
Educational level			
College/university	1,432 (18.1)	296 (10.7)	1,136 (22.2)
High school	4,106 (52.1)	1,392 (50.3)	2,714 (53.0)
Primary school	2,349 (29.8)	1,077 (39.0)	1,272 (24.8)

**Figure 1:**
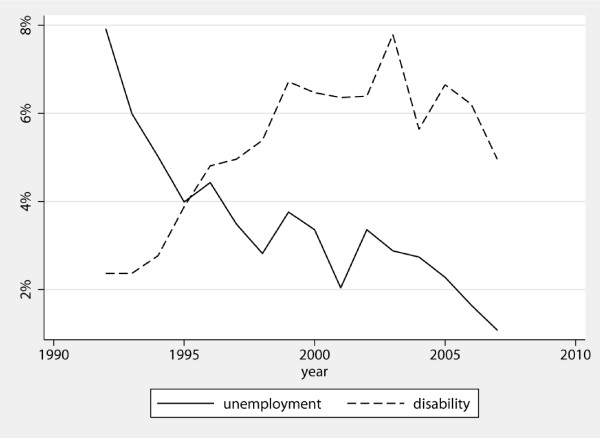
**New unemployment periods and disability pensions per year, 1992-2007 in%**.

Figure 1 shows the per cent of new unemployment periods and disability pensions per year in the cohort during the follow-up period. Within the cohort, there was a decrease of new unemployment periods from 8% in 1992 to 1.1% in 2007. In this period, there was a decline in national unemployment from 5.4% in 1992 to 1.7% at the end of the follow-up period [24].

### Unemployment and disability pension

Table 2 shows the association between unemployment and disability pension within the subsequent year. The odds ratio of unemployment in Model 1 was 1.42 (CI 1.1-1.8). Adjusting for baseline health indicators in Model 2 reduced the estimate to an odds ratio of unemployment to 1.33 (CI 1.1-1.7). Additional adjustment for education further attenuated the odds ratio of unemployment to 1.25 (CI 1.0-1.6) in Model 3.

**Table 2:** The association between unemployment and disability pension.

	Model 1 OR (95% CI)	Model 2 OR (95% CI)	Model 3 OR (95% CI)
Unemployment	1.42 (1.14-1.78)	1.33 (1.06-1.66)	1.25 (1.00-1.56)
Sex (female)	1.58 (1.43-1.74)	1.56 (1.39-1.74)	1.52 (1.36-1.70)
Age in follow-up	1.32 (1.25-1.40)	1.34 (1.26-1.41)	1.34 (1.26-1.41)
Chronic Illness^1^		1.17 (1.11-1.23)	1.17 (1.11-1.23)
Self-rated health: Very good		1.00 (ref)	1.00 (ref)
Good		1.39 (1.21-1.59)	1.35 (1.18-1.54)
Fair		2.08 (1.72-2.50)	2.03 (1.68-2.44)
Poor		3.70 (2.26-6.06)	3.28 (2.00-5.38)
Depressed: Never/rarely		1.00 (ref)	1.00 (ref)
Sometimes		1.10 (0.87-1.39)	1.11 (0.88-1.40)
Often		1.08 (0.85-1.36)	1.08 (0.85-1.37)
Almost all the time		1.14 (0.69-1.87)	1.14 (0.70-1.89)
Headache: Never rarely		1.00 (ref)	1.00 (ref)
Once or several times per month		1.02 (0.91-1.15)	1.03 (0.92-1.16)
Once or several times per week		1.02 (0.85-1.24)	1.02 (0.84-1.23)
Daily		1.35 (0.88-2.06)	1.38 (0.91-2.11)
Pain in neck or shoulder: Never/rarely		1.00 (ref)	1.00 (ref)
Once or several times per month		1.33 (1.18-1.51)	1.31 (1.16-1.48)
Once or several times per week		1.37 (1.16-1.63)	1.32 (1.12-1.58)
Daily		1.90 (1.61-2.24)	1.80 (1.53-2.14)
Smoking: Non-smoker		1.00 (ref)	1.00 (ref)
Former smoker		1.17 (1.01-1.35)	1.11 (0.96-1.20)
Smoker		1.52 (1.34-1.72)	1.38 (1.22-1.98)
Alcohol: Non-drinker		1.00 (ref)	1.00 (ref)
Up to 1-2 times per month		1.09 (0.97-1.22)	1.07 (0.96-1-20)
More than once a week/daily		1.47 (1.15-1.87)	1.55 (1.22-1.98)
Education: High level			1.00 (ref)
Medium level			1.49 (1.27-1.74)
Low Level			2.05 (1.74-2.43)
ICC:	0.02	0.02	0.02
Log likelihood	-7898.4494	-7690.2289	-7649.9913

Discrete time, multilevel regression with one-year time intervals. N = 5,834

^1^A summated index of the number of chronic illnesses described in materials and methods under "health measures"

When the models were tested with a two-year time lag, the odds ratio of unemployment in Model 1 was 1.26 (CI 1.0-1.6) and decreased to 1.17 (CI 0.9-1.5) in Model 2 and to 1.10 (CI 0.9-1.4) in Model 3. When testing for risk of disability the same year as unemployment, the odds ratio was 1.16 (CI 0.9-1.5) in Model 1, 1.08 (CI 0.8-1.4) in Model 2 and 1.02 (CI 0.8-1.3) in Model 3. Having register data on all individuals, Model 1 was also tested including the individuals who did not answer the survey. The odds ratio of unemployment was 1.52 (1.27-1.82). The ICC and the association between sex and age on the risk of disability pension, was the same as in the original model.

There were substantial associations between sex, different measures of poor health, educational level, smoking and alcohol use and disability pension. There was no statistical evidence of effect measure modification between sex and unemployment on disability pension (p-value interaction = 0.55 in the fully adjusted model). The odds ratio of unemployment and disability pension was 1.16 (CI 0.8-1.6) for women and 1.34 (CI 1.0-1.8) for men. There was no evidence of effect measure modification between unemployment and education (p-value = 0.11). The fully adjusted odds ratio of unemployment for people with a low education level was 1.02 (CI 0.7-1.5), compared to 1.54 (CI 1.1-2.1) for people with medium level of education and 0.41 (CI 0.1-3.0) for people with high level of education. There was no support for effect measure modification between unemployment and age in follow-up (p-value = 0.43). The fully adjusted odds ratio (compared to Model 3) of unemployment on receiving disability pension was 1.06 (CI 0.8- 1.5) in the first half of the follow-up period and 1.27 (CI 0.9-1.7) in the last half of the period.

### Differences between municipalities

The multilevel analysis indicates relative small geographical differences in the disability pension risk. The ICC at the municipality level was approximately 2%; however, it was statistically significant, suggesting that the municipality differences were larger than what would be expected due to chance alone.

This result was seen in all the three models. That is, adjusting for compositional differences across municipalities of sex, age, education, health and life style did not influence the ICC estimate.

## Discussion

### Main findings

The main finding in this study was the association between unemployment and disability pension in the subsequent year. This association was attenuated with adjustments for baseline health status, lifestyle and education, suggesting that these factors may act as common causes for both unemployment and disability pension. We found only weak statistical interactions between unemployment and sex, education and age. A minor but significant risk of disability pension can be attributed to individual municipality characteristics.

### Strength and limitations

One of the main strengths of this study was the long follow-up period for the cohort and the high response rate. The study covered a total county population aged 40-42 without disability pension at baseline residing in the same county during the 18-year follow-up period. Although there have been considerable demographical changes in the county, only 6% of the population moved within the county during the follow-up period. Last, the information in this study was obtained from a highly reliable source established by Statistics Norway and the Norway Social Insurance Service.

The study's questionnaire did not contain information from formerly validated health scales. However, we have included comprehensive information on the diseases and complaints that are recognised as risk factors for disability pension. Furthermore, the single item measure of self-rated health is a common measure both for physical and mental health and is also a strong and independent predictor for disability pension [25-27].

The study did not contain information on the reasons that people became unemployed and only measured new unemployment periods. Thus, it does not grasp the difference between becoming unemployed and being unemployed long-term, where the latter likely has a substantial effect on the risk for disability pension. The analysis conducted may also include persons with regular seasonal employment, which may have attenuated the estimate of the risk of disability pension after unemployment.

The regression models were limited to the participants with complete information for all study variables (5,834). There might be selection effects in the study, meaning that the respondents who chose not to answer questions about their health or health behaviour may have a higher or lower risk of being granted disability pension than the other respondents.

Despite the long follow-up time, the legal framework for receiving disability pension has been stable in this period, and thus it is not likely that changing policies have affected this study. In 2004 there was a major policy change when what was called "time-limited disability pension" were introduced, but this affected mainly younger persons, and not the participants of this study, who were then around 55 years of age.

### Unemployment and disability

A recent study from Iceland investigating unemployment and disability pensions from 1992 to 2007 revealed that two large upswings in unemployment had corresponding increases in disability pensions [28]. This suggests that even though health determines the overall incidence of disability pension, marginal fluctuations over time can be related to environmental conditions, like the unemployment rate. When unemployment rates are high, unemployed people with minor health impairments are likely to have more problems finding new jobs, and thus periods of high unemployment rates can lead to more people where work disability arises from the combination of health impairments and poor employment opportunities. The present study's results indicate that the association between unemployment and disability pension could be confounded by health factors. However, it is possible that the association between unemployment and disability pension could be biased according to the presence of time-dependent confounders that are affected by prior unemployment. Hence, further studies are needed that implement longitudinal health measures prior to and after unemployment.

Traditionally, research has suggested that unemployment has stronger negative health effects on men because of gender roles and less financial support from their spouses [29,30]. Two recent meta-analyses summarize the impact of unemployment on physical and psychological well-being reported divergent results. While McKee-Ryan et al. [31] concluded that unemployed women had worse mental health and lower life satisfaction than men, Paul and Moser [29] found that men were substantially more distressed by unemployment than women. A recent study from North Sweden found no support that either gender was more affected by the health consequences of unemployment, and the authors argued that it is less likely to find sex differences in health consequences in Scandinavian countries because of the high female participation in the labour market [30]. In this study, women had a higher risk of receiving disability pension, and although one might assume that women are more often employed in the health services and other public sector professions, which are less influenced by business market fluctuations, this study found weak statistical evidence of gender differences in terms of the likelihood of receiving disability pension after being unemployed.

McKee-Ryan et al. found a u-shaped association where youths and persons older than 50 suffered more from unemployment than middle-aged [31]. Paul and Moser found no clear relationship between age and health outcomes during unemployment [29]. Since we argue that disability pension can be a combination of both health impairments and poor employment opportunities, one might expect that older people, who experience more health problems and possible labour market discrimination, would have a higher risk of receiving disability pensions. Because our study only comprised people from 40 years of age and older, we do not know how our results relate to people of younger age. Despite the association between age and disability pension, we did not find any support that people who became unemployed later in the follow-up period had a higher odds of subsequent disability pension.

Previous research findings suggest that people of high socioeconomic and occupational status have access to better financial and social resources and therefore may be less affected by unemployment. At the same time, these people have lower unemployment rates than people in low-status groups [29]. This study used education as a measure of socioeconomic status, and despite the association between education and disability pension, the results showed only modest support for the impact of educational level on the association between unemployment and disability pension.

### Municipality differences

These findings suggest that the place of residence was of minor importance for the individuals' risk of receiving disability pension. There have been substantial economic and labour market variations between the municipalities in the Nordland County, and a previous descriptive study has shown considerable differences in disability pension incidence rates between the municipalities [32]. With this background, it was expected that the risk of receiving disability pension would be more dependent on municipality residence. However, prior research has not been performed within a multilevel analytic framework, a suitable tool to handle outcomes that are likely to be affected by contextual factors. Nevertheless, the present study's results agree with research on health outcomes that has shown small differences between municipalities using multilevel regression models [33].

Although the municipality is and has been an important contextual level for the local division of government administration (in terms of employment, welfare, health services, etc.), municipalities are diverse when considering their size and inhabitants. Further research should consider other contextual levels, like neighborhoods, economical regions or other levels that may affect the risk of receiving disability pensions. For instance, recent studies have found peer or network effects to be associated with disability pension [34] and welfare participation [35], suggesting that a person's propensity to receive a disability pension can be affected by the disability pension entry rate of similarly-aged workers in his or her neighborhood.

## Conclusions

Numerous studies on unemployment and health outcomes have shown divergent findings, especially relating to age and sex. Although there are substantial associations between sex, age and education and disability pension, this study revealed no or only modest effect modification between unemployment and sex, age and education on the odds of subsequent disability pension. This result indicate that becoming unemployed is only a moderate risk-factor itself. However, if job loss has an effect on health behaviour, this suggests that long-term unemployment can have different effects on older people, who experience more health problems, or on people in the lower social class, who might have poorer health behaviors and coping strategies.

In conclusion, becoming unemployed increased the risk of receiving subsequent disability pension. However, adjusting for baseline health status, health behaviour and education attenuated the impact of unemployment considerably. The multilevel analysis indicated that the geographical differences in disability pension risk were only attributable to municipality characteristics to a minor extent; however, this difference was larger than would be expected by chance alone.

## Competing interests

The authors declare that they have no competing interests.

## Authors' contributions

MS carried out the data processing, the epidemiological modeling andstatistical analysis and wrote the manuscript. KP, RJ and JHB contributed to the epidemiological modeling, statistical analysis, data interpretation and drafting of the manuscript. NF, ES and BC participated in the design of the study and helped to write the manuscript. All authors read and approved the final manuscript.

## Pre-publication history

The pre-publication history for this paper can be accessed here:

http://www.biomedcentral.com/1471-2458/12/148/prepub
